# The Position of (Therapeutical) Damiana

**Published:** 1877-11

**Authors:** 


The Position of (Therapeutical) Damiana.—Commenced in quackish style, damiana has come to a finis easily foreseen. Dr. L. P. Yandell, Jr., of Louisville, tells what he knows about it in a recent paper. He says, "Damiana is almost certainly an unmitigated fraud. Three distinct vegetable products are sold under the name. While it may have produced some very remarkable results, these have been brought about, in all probability, through the imagination of the patient. Could a medicine be discovered possessing the aphrodisiac power attributed to damiana, half of the arable land of the earth would be devoted to its cultivation, and the supply would then not equal the demand."

Good-bye, damiana, and the bright hopes you fostered.—*Medical and Surgical Reporter.*

